# Human papillomavirus disease in *GATA2* deficiency: a genetic predisposition to HPV-associated female anogenital malignancy

**DOI:** 10.3389/fimmu.2024.1445711

**Published:** 2024-08-29

**Authors:** Ehren Dancy, Pamela Stratton, Dominique C. Pichard, Beatriz E. Marciano, Edward W. Cowen, Alison A. McBride, Koenraad Van Doorslaer, Melissa A. Merideth, Noemi Salmeri, Marybeth S. Hughes, Theo Heller, Mark Parta, Dennis D. Hickstein, Heidi H. Kong, Steven M. Holland, Christa S. Zerbe

**Affiliations:** ^1^ Laboratory of Clinical Immunology and Microbiology, National Institute of Allergy and Infectious Diseases, National Institutes of Health, Bethesda, MD, United States; ^2^ Department of Medicine, University of Pennsylvania Perelman School of Medicine, Philadelphia, PA, United States; ^3^ Office of the Clinical Director, National Institute of Neurological Disorders and Stroke, National Institutes of Health, Bethesda, MD, United States; ^4^ National Center for Advancing Translational Sciences, National Institutes of Health, Bethesda, MD, United States; ^5^ Dermatology Branch, National Institute of Arthritis and Musculoskeletal and Skin Diseases, National Institutes of Health, Bethesda, MD, United States; ^6^ Laboratory of Viral Diseases, National Institute of Allergy and Infectious Diseases, National Institutes of Health, Bethesda, MD, United States; ^7^ Department of Immunobiology, College of Medicine, BIO5 Institute, Cancer Biology Graduate Interdisciplinary Program, Genetics Graduate Interdisciplinary Program, University of Arizona Cancer Center, University of Arizona, Tucson, AZ, United States; ^8^ Office of the Clinical Director, National Human Genome Research Institute, National Institutes of Health, Bethesda, MD, United States; ^9^ Gynecology/Obstetrics Unit, Istituto di Ricovero e Cura a Carattere Scientifico (IRCCS) San Raffaele Scientific Institute, Vita-Salute San Raffaele University, Milan, Italy; ^10^ Rehabilitation Medicine Department, Clinical Center, National Institutes of Health, Bethesda, MD, United States; ^11^ Department of Surgery, Eastern Virginia Medical School, Norfolk, VA, United States; ^12^ Liver Diseases Branch, National Institute of Diabetes and Digestive and Kidney Diseases, National Institutes of Health, Bethesda, MD, United States; ^13^ Retired, Bethesda, MD, United States; ^14^ Center for Cancer Research, National Cancer Institute, National Institutes of Health, Bethesda, MD, United States

**Keywords:** *GATA2* haploinsufficiency, human papillomavirus, HPV, high-grade squamous epithelial lesion, HSIL, hematopoietic stem cell transplantation, HSCT or HCT, HPV vaccination

## Abstract

**Objective:**

Patients with pathogenic variants in the GATA Binding Protein 2 (*GATA2*), a hematopoietic transcription factor, are at risk for human papillomavirus-related (HPV) anogenital cancer at younger than expected ages. A female cohort with *GATA2* haploinsufficiency was systematically assessed by two gynecologists to characterize the extent and severity of anogenital HPV disease, which was also compared with affected males.

**Methods:**

A 17-year retrospective review of medical records, including laboratory, histopathology and cytopathology records was performed for patients diagnosed with *GATA2* haploinsufficiency followed at the National Institutes of Health. Student’s *t*-test and Mann-Whitney U test or Fisher’s exact test were used to compare differences in continuous or categorical variables, respectively. Spearman’s rho coefficient was employed for correlations.

**Results:**

Of 68 patients with *GATA2* haploinsufficiency, HPV disease was the initial manifestation in 27 (40%). HPV occurred at median 18.9 (15.2-26.2) years in females, and 25.6 (23.4-26.9) years in males. Fifty-two (76%), 27 females and 25 males, developed HPV-related squamous intraepithelial lesions (SIL) including two males with oral cancer. Twenty-one patients developed anogenital high-grade SIL (HSIL) or carcinoma (16 females versus 5 males, (59% versus 20%, respectively, p=0.005) at median 27 (18.6-59.3) years for females and 33 (16.5-40.1) years for males. Females were more likely than males to require >2 surgeries to treat recurrent HSIL (p=0.0009). Of 30 patients undergoing hematopoietic stem cell transplant (HSCT) to manage disease arising from *GATA2* haploinsufficiency, 12 (nine females, three males) had persistent HSIL/HPV disease. Of these nine females, eight underwent peri-transplant surgical treatment of HSIL. Five of seven who survived post-HSCT received HPV vaccination and had no or minimal evidence of HPV disease 2 years post-HSCT. HPV disease persisted in two receiving immunosuppression. HPV disease/low SIL (LSIL) resolved in all three males.

**Conclusion:**

Females with *GATA2* haploinsufficiency exhibit a heightened risk of recurrent, multifocal anogenital HSIL requiring frequent surveillance and multiple treatments. *GATA2* haploinsufficiency must be considered in a female with extensive, multifocal genital HSIL unresponsive to multiple surgeries. This population may benefit from early intervention like HSCT accompanied by continued, enhanced surveillance and treatment by gynecologic oncologists and gynecologists in those with anogenital HPV disease.

## Graphical Abstract

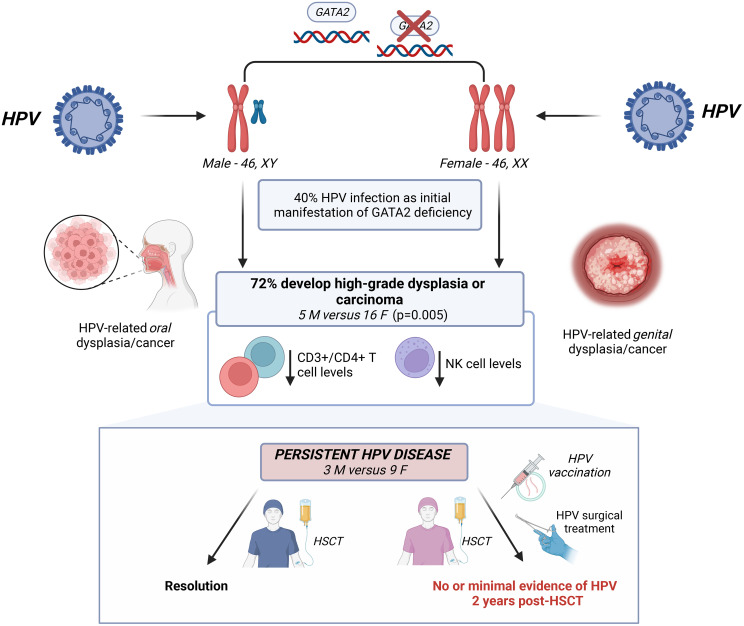

## 1 Introduction

GATA Binding Protein 2 (*GATA2*) haploinsufficiency is an autosomal dominant or sporadically inherited primary immunodeficiency first genetically described in 2011. Nonsense, regulatory, intronic, and missense mutations in *GATA2* lead to clinical manifestations refractory to medical and surgical therapy including human papillomavirus (HPV) leading to high grade squamous intraepithelial lesions (HSIL), hematologic malignancy, and other associated conditions (1, 2). *GATA2* is a transcription factor predominately expressed in early hematopoietic progenitor cells, adult stem cells, and mast cells (3, 4). *GATA2* haploinsufficiency occurs due to inactivation or loss of one copy of *GATA2*, and has been associated with: monocytopenia and nontuberculous mycobacterial (NTM) infections (MonoMac Syndrome), dendritic/monocyte/B/Natural Killer (NK)-cell lymphoid deficiencies (DCML), familial myelodysplastic syndrome, Emberger syndrome, and classical NK cell deficiency (5). Allogeneic hematopoietic stem cell transplantation (HSCT) is curative of the hematologic and immunologic manifestations of *GATA2* haploinsufficiency (6, 7).

HPV disease has been reported in 79% of patients with *GATA2* haploinsufficiency (8). Cutaneous common warts, and oropharyngeal and anogenital disease are prevalent with 35% of patients developing HSIL caused by HPV (5, 9). In patients followed at the National Institutes of Health (NIH) Clinical Center, most *GATA2* haploinsufficient patients had marked monocytopenia, B lymphocytopenia or NK lymphocytopenia, which developed and worsened over time (5, 10).

Patients with primary immunodeficiency often present with phenotypical features typical to each immune dysfunction including HPV infection which underscores the important role the immune system plays in control of HPV disease and enables clinicians to identify particular primary immunodeficiencies. As described by Leiding and Holland, HPV infection that becomes severe and recalcitrant to treatment is common to primary immunodeficiencies such as warts, hypogammaglobulinemia, infections, myelokathexis (WHIM), idiopathic CD4 lymphopenia, dedicator of cytokinesis 8 (DOCK8) deficiency and Emberger syndrome to name a few (11). Although the frequency of severe, genital HPV disease in these immunodeficiencies is not well-established given the rarity overall of these disorders, *GATA2* haploinsufficiency is unique in that HPV disease may be the first or the earliest phenotypic presentation of immune dysfunction.

HPV is a small non-enveloped DNA virus; persistent HPV infection with high-risk types is a risk factor for anogenital and oral malignancy (12–14). HPV is one of the most common sexually transmitted infections among women and men in the United States, with a 22.0% prevalence of disease-associated HPV infection in the sexually active population (15, 16). High risk oncogenic HPV types are associated with >93% of cervical cancers, >80% of vulvar HSIL, >92% of vaginal HSIL, 50% of penile cancers, and 20% of oropharyngeal cancers (17). In the general population, the latency from HPV infection to development of HSIL varies depending on high-risk HPV type (18).

The risk factors for developing persistent HPV disease are influenced by viral (genetic and possibly epigenetic), host, and behavioral factors such as smoking, multiparity, and long-term use of hormonal contraceptives (15); immune competence is critical, especially NK cytotoxicity (19, 20). We hypothesized that individuals with *GATA2* haploinsufficiency might exhibit more pronounced clinical manifestations of HPV disease than the general population, with sex differences between males and females potentially accounting for some variations in clinical manifestations and disease severity.

## 2 Materials and methods

### 2.1 Study participant details

A retrospective review of medical records from May 2000 to February 2017 at the National Institutes of Health (NIH) was performed to identify patients with known *GATA2* mutations, including clinical notes, patient questionnaires, laboratory, histopathology, and cytopathology records. All patients provided written informed consent and were enrolled on protocols approved by an NIH IRB in which the natural history of *GATA2* mutations was studied (ClinicalTrials.gov Identifier: NCT01905826, NCT00018044, NCT00404560).

All patients with known *GATA2* haploinsufficiency enrolled in these natural history studies were included. Their charts from the NIH Clinical Center’s electronic medical record were retrospectively reviewed for symptoms commonly observed with *GATA2* haploinsufficiency. Information about time of onset and disease progression for common cutaneous and anogenital warts, hematologic abnormalities, infections (including nontuberculous mycobacterial, fungal and non-HPV viral infections), myelodysplastic syndrome, and allogeneic HSCT and post-HSCT HPV disease were summarized. Values for NK cells, CD3+/CD4+ T-helper cells and monocytes from the most recent visit or immediately prior to HSCT for those who underwent HSCT, were abstracted. In general, the female patients over age 18 were systematically examined and assessed by one of 2 gynecologists (PS and MM) and included review of outside gynecology records. Male patients with anogenital HPV disease and female patients with perianal and anal HPV disease were assessed by a single general surgeon (MH) and gastroenterologist (TH). This report includes a subset of a larger published cohort (8) and unpublished data narrowed to the time period in which female subjects were evaluated based on the standard examination by the aforementioned gynecological team. For subjects for whom only the year was provided as the date of diagnosis, the month and date were assigned as June 15^th^.

### 2.2 HPV infection

HPV infection was identified based on clinical, pathologic or laboratory evidence and its location. Extensive oropharyngeal or anogenital HPV disease was defined as multifocal disease that, in the lower genital tract spanned multiple sites such as vulvar and vaginal, vulvar and cervical, or scrotal and inguinal. Severity of HPV disease was categorized as follows: mild HPV disease (localized genital or common cutaneous in the absence of histologically confirmed severe dysplasia; responsive to local treatments); severe HPV disease (multifocal anogenital or oropharyngeal HPV disease, histologically confirmed severe dysplasia to carcinoma *in situ*; high-grade lesions difficult to treat and non-responsive to multiple local treatments). Pathological grade was determined by evidence of low grade squamous intraepithelial lesions (LSIL), HSIL or malignancy on histopathology. Laboratory confirmation of HPV infection was determined by HPV DNA detection. HPV typing from cervical or anal swabs was performed commercially using hybrid capture pooled for high and low risk types (Quest Diagnostics Nichols Institute, Chantilly, VA).

### 2.3 HPV genotyping from lesional tissue

Clinical biopsies obtained from some subjects receiving care at the NIH Clinical Center were genotyped for HPV as previously described (21). One µl of extracted DNA was amplified using the SPF10 primer set (22). PCR amplicons were cloned into the TOPO-TA cloning kit (ThermoFisher). Ten to 22 clones were isolated and sequenced using the M13F and M13R primers. Primer sequences used are shown in **Supplementary Table 1**.

### 2.4 HPV post-HSCT

Resolution and persistence of HPV disease were described at 2 years posttransplant based on pretransplant history. Peri-HSCT and post-HSCT management and clinical examination findings at the most recent post-HSCT follow-up are described. Some subjects underwent treatment of pre-malignant anogenital lesions in the six months prior to transplant and others had treatment delayed until soon after HSCT.

### 2.5 Statistical analysis

Shapiro-Wilk test was employed to assess the normality of the data distribution. The Student’s *t*-test was used to compare differences in continuous normally distributed variables, which are presented as mean ± standard deviation (range). The Mann-Whitney U test was used to compare differences in continuous not normally distributed variables, which are presented as median (interquartile range, IQR). Fisher’s exact test was employed to compare differences in categorical variables, which are reported as absolute values (%). Comparisons between groups were based on biological sex. Immunologic values were stratified into quartiles given the nonparametric distribution of immunologic laboratory values. Spearman’s rho coefficient was employed as a nonparametric measure of the correlation between immunologic laboratory values and the incidence and severity of HPV disease. Correlation coefficients were interpreted as follows: 0.00-0.19 as very weak, 0.20-0.39 as weak, 0.40-0.59 as moderate, 0.60-0.79 as strong, and 0.80-1.00 as very strong (23). Positive and negative values were interpreted as positive or negative correlations, respectively. STATA version 18 software (Stata Corp LLC, 2021, College Station, TX, USA) was used for all statistical analyses. p values <0.05 were considered significant.

## 3 Results

We studied 68 patients with *GATA2* haploinsufficiency followed at the NIH. The median age of onset of *GATA2* haploinsufficiency signs and symptoms in this cohort was 15.9 years in females (9.9 – 23.7 years), and 13.8 years in males (11.2 – 22.9 years). HPV disease was the initial manifestation of *GATA2* haploinsufficiency in 27 of 68 (40%) patients. Age at first HPV manifestation was 18.9 in females (15.2-26.2 years), and 25.6 years in males (23.4-26.9 years). Thirty-four of 68 (50%) patients were infected with a nontuberculous mycobacteria infection and 39 of 68 (56%) progressed to myelodysplastic syndrome.

### 3.1 HPV infection and disease manifestations

**Figure 1:**
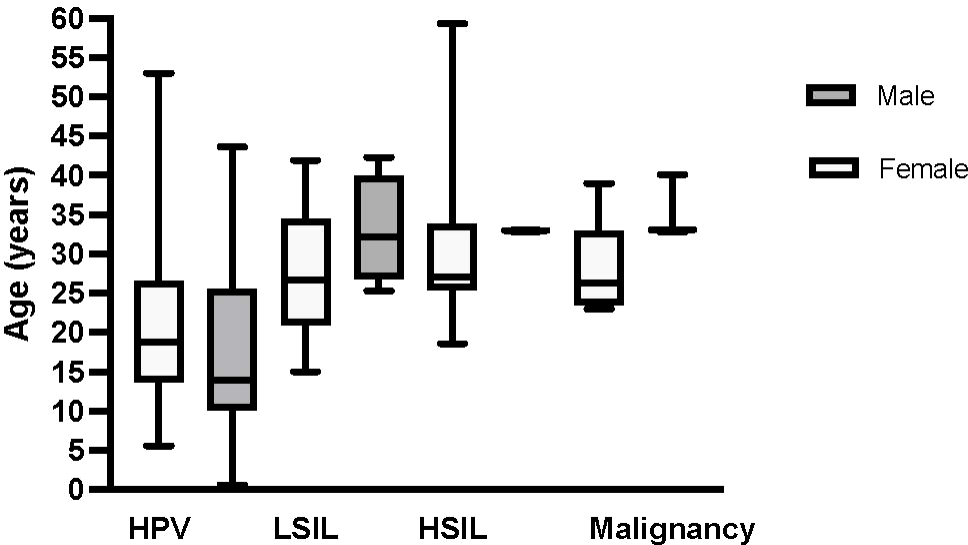
Median age of HPV disease diagnosis in female and male patients with *GATA2* haploinsufficiency. The y-axis represents the age, and the x-axis shows HPV disease progression to cancer to include initial HPV disease diagnosis, low-grade squamous intraepithelial lesion (LSIL), high-grade squamous intraepithelial lesion (HSIL), and HPV-related malignancy that includes either carcinoma *in situ* or carcinoma. Box plots depict females in light grey and males in dark grey. Median age and range by sex for each HPV disease category is as follows: HPV at 19 (6 – 53) years and 14 (3 – 40) years in females and males, respectively, LSIL at 27 (15 – 42) years and 32 (25 – 33) years in females and males, respectively, HSIL at 27 (18 – 59) years and 33 (33 – 40) years in females and males, respectively and malignancy at 33 (23 – 59) years and 36 (33 – 40) years in females and males, respectively.

HPV disease was reported in a total of 52 of 68 patients, 27 of 36 (75%) females and 25 of 32 (78%) males (**Table 1**, **Figure 1**). Twenty-one patients developed anogenital HSIL or carcinoma (16 females versus 5 males, (59% versus 20% of those affected by HPV, respectively, p=0.005). The mean time from initial HPV diagnosis to first HSIL in patients with *GATA2* haploinsufficiency was 9.36 ± 7.8 years (range: at first clinical evaluation to 23 years), 8.96 ± 7.68 years (range: 0 – 23.9) in females compared to 10.65 ± 9.26 years (range: 2.5 – 23) for males (p = 0.717). The majority of patients developed HPV-associated HSIL by their early 30s, regardless of biological sex (median age in females: 27 years (range: 18.6-59.3), in males: 33 years (range: 16.5-40.1), **Figure 1**). In the 10 patients under age 20 (5 males, 5 females), only one male had evidence of genital HPV disease. Seventeen patients developed oral (2 males) or anogenital carcinoma or carcinoma *in situ* (2 males; 13 females). The two cases with oropharyngeal cancer were successfully treated with medical and surgical treatment. Sixteen female patients had severe, multifocal anogenital HPV with the most frequent areas of involvement as vulvar (n=15) and cervical (n=13) sites. Surgical management for HPV/HSIL/cancer was reported in 24 patients. Females were more likely than males to undergo medical or surgical treatment of HPV/HSIL (19/20 females versus 5/12 males p=0.0006). In addition, a greater number of females were significantly more likely than males to need more than 2 surgical treatments to treat recurrent disease (range 3 to 20+ procedures, 12/19 females versus 1/5 males p=0.0009). Surgical treatment varied depending on anatomic location. Cervical HSIL was treated by cone biopsy, loop electrocautery excision procedure, or laser ablation of the cervix. Vaginal HSIL was treated by surgical laser ablation of lesions. Vulvar and other external anogenital HSIL was treated with surgical excision and laser or plasma-jet ablation of lesions. Five female patients underwent vulvectomies, of which two required skin grafting. One vulvectomy patient required a temporary diverting colostomy and one required co-incident total hysterectomy.

**Table 1:** Characterization of HPV disease spectrum in the *GATA2* haplodeficient population.

	Total (n=52)	Female (n=27)	Male (n=25)	p-value^a^
Location				
**Cutaneous/extragenital warts**	40	17	23	0.02
**Oral/tracheal HPV**	2	0	2	0.22
**Anogenital tract HPV overall**	32	20	12	0.09
**Anogenital tract HPV location^b^**				n/a^c^
Vulva (female)/scrotum, inguinal (male)	19	14	5	
Cervical, vaginal (female)/penile (male)	22	14	8	
Anal	7	4	3	
**Anogenital Squamous Intraepithelial Neoplasia requiring treatment**	25	19	6	0.001
i) Grade				0.005
LSIL	4	3	1	
HSIL	21	16	5	
(*in Situ*/Malignant)	(12)	(9)	(3)	
ii) Treatment				
Medical	2^d^	1^d^	1	
Surgical				0.0009
1-2	11^d^	7^d^	4	
> 3	13	12	1	
iii) HSCT				0.40
Total	20	12	8	
HPV disease at time of transplant	12	9	3	
**HPV vaccination**	11	11	n/r^e^	n/a^c^

^a^Fisher’s exact text comparing male and female cohorts; ^b^Multiple locations common; ^c^n/a is not appropriate for comparison; ^d^both medical and surgical approach in 1 patient; ^e^n/r is not recorded in medical records. HPV, human papillomavirus; LSIL, low-grade squamous intraepithelial lesion; HSIL, high-grade squamous intraepithelial lesion; HSCT, hematopoietic stem-cell transplantation.

HPV genotype was identified by PCR on 9 histological specimens obtained at NIH of which 8 were vulvar and 1 was cervical (**Table 2**). HPV-16 and -18 were identified in 6 of 9 lesions. Two HSIL vulvar specimens only expressed HPV-66, a weakly carcinogenic HPV type and one LSIL cervical specimen expressed HPV-90 (23, 24). Three different HPV types were identified in two separate samples.

**Table 2:** HPV genotypes in female genital HPV-related squamous epithelial or carcinoma lesions.

Age in years: first HPV infection/current age	Location of lesion	Clinical diagnosis	HPV types detected
22/39	Vulva	SCC in-situ	16
18/43	Vulva	VIN 3	18, 33, 56
18/43	Vulva	High grade squamous intraepithelial neoplasia, VIN2 and VIN3	16
18/23	Vulva	Invasive SCC, moderately differentiated	16
21/24	Vulva	VIN 2	66
17/22	Vulva	VIN 3	16
16/28	Vulva	High grade squamous intraepithelial lesions	66
24/32	Cervix	Low-grade squamous intraepithelial lesions (CIN 1)	90
25/28	Vulva	VIN 2	18, 90, 6

SCC *in-situ*, squamous cell carcinoma *in-situ*; VIN 3, vulvar intraepithelial neoplasia grade 3; VIN 2, vulvar intraepithelial neoplasia grade 2; CIN 1, cervical intraepithelial neoplasia grade 1.

We then sought to analyze immunologic subtype values and HPV disease incidence and severity in the overall cohort and in sex-specific cohorts (**Supplementary Table 2**). Prior to transplant across the whole cohort, there were positive correlations among the various immune cell lineages, specifically among NK cell, CD3+/CD4+ T cell, and monocyte levels. Notably, higher CD3+/CD4+ T cell levels were negatively correlated with HPV incidence (rs=-0.339, p=0.008), while higher NK cell levels were negatively correlated with both HPV incidence (rs=-0.377, p=0.002) and severity (rs=-0.3151, p=0.0136). In sex-specific quartiles, higher NK cell levels in males were negatively correlated with HPV presence (rs=-0.397, p=0.033), while higher CD3+/CD4+ T cell levels were negatively associated with both HPV incidence (rs=-0.383, p=0.039) and severity (rs=-0.415, p=0.024). No significant correlations were observed between female-specific immune cell quartiles and HPV presence or severity.

### 3.2 HPV and HSCT

Thirty patients (18 females and 12 males) underwent HSCT. Nine of 18 females had anogenital HSIL prior to transplant with seven having undergone more than four surgical treatments. Five of nine females underwent surgical treatment prior to and three others after HSCT.

All females underwent anogenital assessment post-HSCT for evidence of HPV disease. Of the seven females who survived 2 years post-HSCT, five had no or minimal evidence of persistent/recurrent HPV disease. These five females all received HPV vaccination as part of routine post-transplant care. The two others who continued immunosuppression did not undergo HPV vaccination. Three of the 12 males who underwent HSCT had HPV disease prior to transplant involving penis, anal, or oral cavity. All three males experienced resolution of their LSIL HPV disease after HSCT without surgical intervention.

## 4 Discussion

### 4.1 Summary of main results

*GATA2* haploinsufficiency includes early-onset multifocal, recurrent, HPV-related genital squamous intraepithelial neoplasia and carcinoma, disproportionately affecting females and frequently involving both the vulva and cervix. Multilineage cytopenias involving NK cells, B cells and monocytes are common and characteristic of *GATA2* haploinsufficiency, as is progression to myelodysplastic syndrome and acute myeloid leukemia, which suggests early HSCT would be beneficial (5–7). Importantly, in 70% of our patients, severe infection, including HPV, was the initial presentation of *GATA2* haploinsufficiency (2). We cannot determine whether NK or T cells are the critical elements in the development of severe, chronic HPV disease in *GATA2* haploinsufficiency, as both were more depleted in HPV-affected than non-affected individuals; the lack of association with monocyte numbers suggest that monocytes are not the critical elements in HPV disease. A report of a patient with a pathogenic germline mutation in the interleukin-2 receptor subunit gamma gene demonstrated that restoring natural killer cell function with HSCT facilitated treatment of relapsing HPV disease (20).

HPV morbidity in *GATA2* haploinsufficiency is high. Female patients undergo extensive, disfiguring, surgical excisions at young ages including hysterectomy and vulvectomy for HSIL or malignant disease. These procedures, especially vulvectomy, can have harmful effects on mental health and sexual function (25). Undergoing complex surgery increases the risk of complications including surgical diversions related to anogenital malignancies. Female patients with multifocal anogenital sites of HPV disease or those requiring several surgical interventions for HPV must warrant evaluation for *GATA2* haploinsufficiency.

Characterizations of HPV types responsible for dysplasia or carcinoma in immunodeficiency are limited to case reports (26–30). HPV typing performed in 9 of 27 affected females identified high risk HPV types, HPV-16 and HPV-18 in female genital samples. This finding and the observation that only one subject under age 20 had genital HPV disease underscores the potential to boost immune response to HPV infection in order to potentially prevent severe disease by routine HPV vaccination during adolescence, prior to initiation of sexual activity (31).

In the thirty patients who underwent HSCT, twelve patients had HPV disease: nine females with HPV-associated anogenital HSIL and three males with anogenital LSIL. These female patients underwent systematic, comprehensive examinations of the genital and anal sites for HSIL/HPV disease followed by treatment of HSIL. All but two females with a history of recurrent vulvar and cervical HSIL experienced resolution of HPV disease after HSCT by two years with peritransplant surveillance and management. In a recent paper describing outcome of HPV infection in *GATA2* haploinsufficiency post-HSCT, post-HSCT monocyte or NK cell numbers were not correlated with HPV outcome, suggesting that active management (comprised of surveillance and surgery by a gynecologic and gastrointestinal team) likely contributed to lesion resolution (8). Additionally, HPV vaccination after transplant appears to be safe and may be effective in generating immunity (32). As HPV vaccination is not therapeutic, it is unlikely that HPV vaccination after HSCT contributed to resolution of disease in these 5 females.

### 4.2 Results in the context of published literature

Other primary immunodeficiencies, such as Dedicator Of Cytokinesis 8 (DOCK8) deficiency, Warts, Hypogammaglobulinemia, Infections, and Myelokathexis (WHIM) syndrome, and Epidermodysplasia Verruciformis are associated with HPV disease, implying that multiple mechanisms are involved in the control of HPV infection (11). Studies on HPV disease in HIV suggest that low CD4 T-cell counts alone do not account for refractory HPV disease (19, 33, 34). The importance of NK cells in viral immunity, including against HPV infection, is well established (35). Type I interferons activate NK cells, which directly kill infected cells and produce pro-inflammatory cytokines.

Patients with *GATA2* haploinsufficiency have dysfunctional as well as low numbers of NK cells (36). We found a significant negative correlation between NK cell and CD3+/CD4+ T cell levels and both the incidence and severity of HPV disease for the whole cohort that persisted in males when stratified by sex-specific quartiles. This association was not observed in females, likely due to the small number of females without severe HPV disease. The reduction in NK number and function likely contributes to the recalcitrant nature of HPV infection in *GATA2* haploinsufficiency, similar to the pattern that has been described for severe EBV infection (37).

### 4.3 Strengths and weaknesses

Importantly, when immune reconstitution in *GATA2* haploinsufficient patients was examined over time in an NIH HSCT cohort from just before through survival after HSCT, HPV disease was controlled and resolved (8). In this study, the systematic collection of immune features at one time point minimized ascertainment and recall bias. However, examination of the interplay between immunological parameters and HPV disease over the life course was not possible and is a limitation. Detailed immunological profiles obtained within this study were not measured as part of routine gynecologic care prior to study participation. Longitudinal analysis is further complicated by the individual variability inherent in the unpredictable time between occurrence of HPV infection, disease and diagnosis as disease onset is generally asymptomatic, except when lesions become extensive. This variability in disease presentation undoubtedly contributes to ascertainment and recall bias regarding the timing of development of disease. The need for multiple surgeries that are unsuccessful in controlling HSIL may lead to underreporting of surgical procedures and recall bias. Despite these limitations, the ability to document the severity and recurrence of HPV disease in affected females and the lack of HPV disease recurrence in males suggests these findings may be generalizable to other cohorts of *GATA2* affected patients.

At the time of this study, we systematically collected information on HPV vaccination in female patients but did not routinely ask male patients. Not collecting it on male patients is a study limitation. As a result of this work on HPV, we have changed clinical practice within our studies of *GATA2* haploinsufficiency. We now ask all study participants about their HPV vaccination status. Additionally, we vaccinate anyone who has not received HPV vaccination, regardless of their biological sex.

### 4.4 Implications for practice and future research

The extent and morbidity of genital HPV disease in this large cohort, especially among female patients, identifies another genetic predisposition to HPV-associated malignancy. This heightened risk is like that observed in females with other conditions with compromised immune response (38). *GATA2* haploinsufficient women affected by HPV should undergo more frequent cervical cancer screening as recommended for these other populations (38).

The occurrence of severe, recurrent HPV disease beyond the cervix, across the lower genital tract, underscores some unique aspects of gynecologic screening in *GATA2* haploinsufficient females. In particular, the heightened risk of vulva cancer we observed among females in their 20s in this cohort study underscores the importance of comprehensive lower genital tract assessment undertaken as part of routine care and continuing across their reproductive life. Colposcopy of the vulva, vagina, or cervix should be performed when any abnormal areas are seen on visual inspection or if a cervical cytology test is abnormal. Lesions identified during colposcopy or routine examination should be biopsied promptly to identify HSIL needing treatment prior to development of cancer. Among those identified with HSIL, continued frequent assessment every six months or so, is critically important to preventing or identifying cancer at its earliest stages.

This cohort study suggests that routine HPV vaccination should be implemented early, prior to acquiring HPV infection and to take advantage of intact immune function early in life. The effectiveness of HSCT on immune reconstitution for the resolution of HSIL suggests that anogenital HPV disease itself may be an important consideration in the decision to undergo HSCT. Comprehensive evaluation for anogenital HPV-related disease is warranted before HSCT and should continue post-HSCT so that surgical and other therapeutic measures can be undertaken in those with new HSIL or persistent disease. Most importantly, these data confirm that recurrent or multifocal HSIL should prompt consideration of *GATA2* haploinsufficiency as the underlying cause.

The therapeutic challenges in treatment of severe HPV disease using surveillance and standard surgical approaches illustrate important research opportunities in *GATA2* haploinsufficient females. Identification of genetic mutations causative in GATA2 haploinsufficiency across familial generations could identify those at risk for developing HPV disease and prompt early intervention and surveillance by gynecologists. Such identification could then allow examination of whether prophylactic HPV vaccination alone or in combination with HSCT are effective in preventing HSIL or persistent disease in *GATA2*. Females with *GATA2* haploinsufficiency are a potential cohort for study of therapeutic HPV vaccination once available.

### 4.5 Conclusion

Female patients with *GATA2* haploinsufficiency exhibit a heightened risk for severe, extensive, difficult to treat HPV disease and develop anogenital cancer at younger than expected ages compared to the general population. Accordingly, in females with extensive, multifocal genital HSIL unresponsive to multiple surgeries, as demonstrated by this study, *GATA2* haploinsufficiency must be considered. Therefore, gynecologic oncologists and other women’s health practitioners could and should play an important role in early identification of rare immunodeficiencies when treating severe, multifocal, refractory anogenital HPV disease. Subsequent ability to effectively manage recurrent vulvar and cervical HSIL with surgery in patients with *GATA2* haploinsufficiency who underwent HSCT suggests that restoration of normal immune function alongside anogenital surveillance and treatment together embody the successful approach to controlling HPV disease in this population.

## Data Availability

The raw data supporting the conclusions of this article will be made available by the authors, without undue reservation.

## Author disclaimer

The opinions expressed within the content are solely the author’s and do not reflect the opinions of the National Institute of Allergy and Infectious Diseases, National Institute of Neurological Disorders and Stroke, National Institutes of Health Clinical Center, National Cancer Institute, National Center for Advancing Translational Sciences, National Human Genome Research Institute, National Institute of Diabetes and Digestive and Kidney Diseases, and the National Institute of Arthritis and Musculoskeletal and Skin Diseases at the National Institutes of Health of the United States Government.
