# Supplementary material for: Human papillomavirus disease in GATA2 deficiency: a genetic predisposition to HPV-associated female anogenital malignancy
Source: Front Immunol. 2024 Aug 29;15:1445711. doi: 10.3389/fimmu.2024.1445711 (PMC11390362; doi:10.3389/fimmu.2024.1445711)
Supplement: Supplementary file 1 [file Table1.docx]

**Supplementary Table 1.** Primers utilized in genotyping.

|  | **Sequence (5’-3’)** | **Reference** |
| --- | --- | --- |
| **SPF1A** | GCiCAGGGiCACAATAATGG | (Quint et al., 2001) |
| **SPF1B** | GCiCAGGGiCATAACAATGG | (Quint et al., 2001) |
| **SPF1C** | GCiCAGGGiCATAATAATGG | (Quint et al., 2001) |
| **SPF1D** | GCiCAAGGiCATAATAATGG | (Quint et al., 2001) |
| **SPF2A** | GTiGTATCiACAACAGTAACAAA | (Quint et al., 2001) |
| **SPF2B** | GTiGTATCiACTACAGTAACAAA | (Quint et al., 2001) |

“i” represents inosine, will base pair with A, C or T
