# Supplementary material for: Human papillomavirus disease in GATA2 deficiency: a genetic predisposition to HPV-associated female anogenital malignancy
Source: Front Immunol. 2024 Aug 29;15:1445711. doi: 10.3389/fimmu.2024.1445711 (PMC11390362; doi:10.3389/fimmu.2024.1445711)
Supplement: Supplementary file 2 [file Table2.docx]

**Supplementary Table 2**. Correlation matrices between overall and sex-specific Natural Killer (NK) cells, CD3+/CD4+ T-Helper cells and monocytes immune cell values expressed in quartiles and incidence and severity of HPV disease in the entire *GATA2* haploinsufficient patient cohort.

a) Overall cohort (n=61)

|  | HPV disease | Severe HPV | NK cells | CD3+/CD4+ | Monocytes |
| --- | --- | --- | --- | --- | --- |
| HPV disease | 1 | 0.762**** | -0.377** | -0.334** | -0.165 |
| Severe HPV | 0.762**** | 1 | -0.315* | -0.231 | -0.079 |
| NK cells | -0.377** | -0.315* | 1 | 0.646**** | 0.435*** |
| CD3+/CD4+ | -0.334** | -0.231 | 0.646**** | 1 | 0.306* |
| Monocytes | -0.165 | -0.079 | 0.435*** | 0.306* | 1 |

*: p<0.05; **: p<0.01; ***: p<0.001; ****: p<0.0001.

b) Male cohort (n=29)

|  | HPV disease | Severe HPV | NK cells | CD3+/CD4+ | Monocytes |
| --- | --- | --- | --- | --- | --- |
| HPV disease | 1 | 0.835**** | -0.397* | -0.383* | -0.239 |
| Severe HPV | 0.835**** | 1 | -0.359 | -0.415* | -0.073 |
| NK cells | -0.397* | -0.359 | 1 | 0.697*** | 0.686*** |
| CD3+/CD4+ | -0.383* | -0.415* | 0.697*** | 1 | 0.534** |
| Monocytes | -0.239 | -0.073 | 0.686*** | 0.534** | 1 |

*: p<0.05; **: p<0.01; ***: p<0.001; ****: p<0.0001.

c) Female cohort (n=32)

|  | HPV disease | Severe HPV | NK cells | CD3+/CD4+ | Monocytes |
| --- | --- | --- | --- | --- | --- |
| HPV disease | 1 | 0.768**** | -0.182 | -0.245 | 0.017 |
| Severe HPV | 0.768**** | 1 | -0.166 | -0.038 | -0.013 |
| NK cells | -0.182 | -0.166 | 1 | 0.478*** | 0.416* |
| CD3+/CD4+ | -0.245 | -0.038 | 0.478*** | 1 | 0.096 |
| Monocytes | 0.017 | -0.013 | 0.416* | 0.096 | 1 |

*: p<0.05; **: p<0.01; ***: p<0.001; ****: p<0.0001.

In each matrix, the rows and columns represent the variables being correlated. Each cell in the matrix displays the calculated Spearman’s Rho correlation coefficient. Values range between -1 and 1, indicating the strength and direction of the relationship between two variables: a positive value signifies a positive correlation, while a negative one denotes a negative correlation. Immune cell levels stratified into quartiles are presented as aggregated data for the overall cohort and disaggregated data for sex-specific cohorts.
